# *Spdef* deletion rescues the crypt cell proliferation defect in conditional *Gata6* null mouse small intestine

**DOI:** 10.1186/1471-2199-15-3

**Published:** 2014-01-28

**Authors:** Boaz E Aronson, Kelly A Stapleton, Laurens ATM Vissers, Eva Stokhuijzen, Hanneke Bruijnzeel, Stephen D Krasinski

**Affiliations:** 1 Division of Gastroenterology and Nutrition, Department of Medicine, Children’s Hospital Boston, and Harvard Medical School, 300 Longwood Avenue, Boston, MA 02115, USA; 2 Academic Medical Center Amsterdam, Emma Children’s Hospital, Amsterdam, the Netherlands; 3 University Medical Center Groningen, Groningen, the Netherlands

**Keywords:** GATA6, SPDEF, Crypt cell proliferation, Intestinal differentiation

## Abstract

**Background:**

GATA transcription factors are essential for self-renewal of the small intestinal epithelium. *Gata4* is expressed in the proximal 85% of small intestine while *Gata6* is expressed throughout the length of small intestine. Deletion of intestinal *Gata4* and *Gata6* results in an altered proliferation/differentiation phenotype, and an up-regulation of SAM pointed domain containing ETS transcription factor (*Spdef*), a transcription factor recently shown to act as a tumor suppressor. The goal of this study is to determine to what extent SPDEF mediates the downstream functions of GATA4/GATA6 in the small intestine. The hypothesis to be tested is that intestinal GATA4/GATA6 functions through SPDEF by repressing *Spdef* gene expression. To test this hypothesis, we defined the functions most likely regulated by the overlapping GATA6/SPDEF target gene set in mouse intestine, delineated the relationship between GATA6 chromatin occupancy and *Spdef* gene regulation in Caco-2 cells, and determined the extent to which prevention of *Spdef* up-regulation by *Spdef* knockout rescues the GATA6 phenotype in conditional *Gata6* knockout mouse ileum.

**Results:**

Using publicly available profiling data, we found that 83% of GATA6-regulated genes are also regulated by SPDEF, and that proliferation/cancer is the function most likely to be modulated by this overlapping gene set. In human Caco-2 cells, GATA6 knockdown results in an up-regulation of *Spdef* gene expression, modeling our mouse *Gata6* knockout data. GATA6 occupies a genetic locus located 40 kb upstream of the *Spdef* transcription start site, consistent with direct regulation of *Spdef* gene expression by GATA6. Prevention of *Spdef* up-regulation in conditional *Gata6* knockout mouse ileum by the additional deletion of *Spdef* rescued the crypt cell proliferation defect, but had little effect on altered lineage differentiation or absorptive enterocytes gene expression.

**Conclusion:**

SPDEF is a key, immediate downstream effecter of the crypt cell proliferation function of GATA4/GATA6 in the small intestine.

## Background

The mature mammalian small intestine is lined by a highly specialized epithelium that regenerates itself in a tightly controlled manner resulting in a lineage distribution and gene expression patterning that is perfectly suited for the absorption of nutrients. The epithelium is organized into crypt-villus structures in which the Crypts of Lieberkühn contain stem cells that produce proliferating, transit-amplifying (TA) cells that differentiate into five principal post-mitotic cell types comprised of one type of absorptive cell (absorptive enterocytes) and four types of secretory cells (enteroendocrine, goblet, Paneth, and tuft cells [1]). Absorptive enterocytes express digestive enzymes and transporters necessary for the terminal digestion and absorption of nutrients. Mucus-secreting goblet cells and defensin-secreting Paneth cells maintain a dynamic mucosal defensive barrier. Enteroendocrine cells secrete hormones that regulate gastrointestinal processes. Tuft cells, recently shown to be an independent secretory lineage [2], secrete opioids and produce enzymes that synthesize prostaglandins, suggesting a role in inflammation. Absorptive enterocytes, goblet cells, enteroendocrine cells, and tuft cells migrate up the crypt to populate the villi, whereas Paneth cells migrate to the base of crypts. The differentiated cells eventually undergo apoptosis and are shed into the lumen. Cells of the villus epithelium turn over in 3–4 days, whereas Paneth cells at the base of crypts turn over at a slower rate of 3–6 weeks.

Current models of intestinal epithelial renewal suggest that long-lived, multipotent stem cells produce progenitors that undergo a series of transitions that ultimately give rise to the individual cell lineages [1,3]. The Wnt, hedgehog, and bone morphogenetic protein signaling pathways regulate intestinal proliferation and differentiation, while the Notch signaling pathway plays a central role in determining epithelial cell fate. The first decision selects absorptive vs. secretory progenitors. Activated Notch signaling results in the transcriptional activation of its principal intestinal target, hairy and enhancer of split 1 (*Hes1*), which encodes a transcription factor that selects the absorptive enterocyte lineage. Progenitor cells that escape Notch signaling and activation of *Hes1* gene transcription express atonal homolog 1 (*Atoh1,* formerly called *Math1*), which encodes a transcription factor that selects the secretory cells. Additional regulators that function in secretory cell differentiation include: growth factor independent 1 (*Gfi1*) that distinguishes enteroendocrine from goblet/Paneth progenitors; neurogenin 3 (*Neurog3*) that specifies the enteroendocrine lineage; SAM pointed domain-containing Ets transcription factor (*Spdef*), a GFI1 target, that promotes goblet differentiation; and SRY-box containing gene 9 (*Sox9*) and ephrin type B receptor 3 (*Ephb3*), Wnt targets that are necessary for the differentiation of Paneth cells and their localization to the crypt base, respectively.

Recently, we showed that members of the GATA family, an ancient family of transcription factors that bind WGATAR motifs in DNA, play essential roles in crypt cell proliferation, secretory cell differentiation, and absorptive enterocyte gene expression. *Gata4* and *Gata6* are expressed in the intestinal epithelium, but whereas *Gata6* is expressed throughout the length of the small intestine, *Gata4* is expressed in the proximal 85% of small intestine and is sharply down-regulated in the distal ileum [4-6]. Using conditional knockout technology, we [5,7] and others [8] have shown that GATA4 functions to promote a 'jejunal’ pattern of absorptive enterocyte gene expression and function while repressing an 'ileal’ pattern. Using single and double conditional knockout approaches for *Gata4* and *Gata6*, we found that in the ileum of single *Gata6* conditional knockout mice, where *Gata4* is not normally expressed, or throughout the small intestine of double *Gata4/Gata6* conditional knockout mice, crypt cell proliferation and enteroendocrine cell specification are decreased, Paneth cells are replaced by a goblet-like cell type, and the expression of specific absorptive enterocyte genes is altered [4]. We also noted that *Spdef*, a transcription factor expressed in secretory progenitors, goblet cells and Paneth cells that functions in goblet and Paneth cell differentiation [9,10], was up-regulated [4]. Using a conditional *Spdef* over-expression model, Noah et al. [10] described an intestinal phenotype that, with the exception of the changes in absorptive enterocyte gene expression, essentially phenocopies that of our *Gata4/Gata6* conditional knockout mice: crypt cell proliferation is decreased, enteroendocrine and Paneth cells are decreased, and goblet-like cells are increased. GATA6 is co-expressed with SPDEF in the same lineages in the small intestine [4]. Based on these findings [4], we hypothesized that GATA4/GATA6 regulates crypt cell proliferation and secretory cell differentiation in the small intestine by repressing *Spdef* gene expression. To test this hypothesis, we defined the functions most likely regulated by the overlapping GATA6/SPDEF target gene set in mouse intestine, delineated the relationship between GATA6 chromatin occupancy and *Spdef* gene regulation in Caco-2 cells, and determined the extent to which prevention of *Spdef* up-regulation by *Spdef* knockout rescues the GATA6 phenotype in conditional *Gata6* knockout mouse ileum.

## Results and discussion

### GATA6 and SPDEF regulate similar subsets of genes

**Figure 1:**
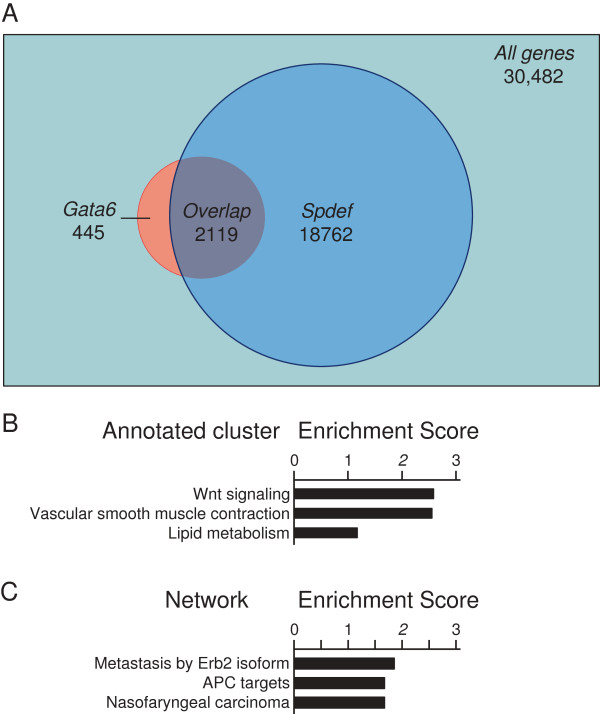
**Overlap of genes in small intestine modulated by conditional *Gata6 *or *Spdef *knockout. (A)** Publicly available gene profiling data from conditional *Gata6* (GSE22416) and *Spdef* (GSE14892) knockout mouse intestine show a significant overlap. **(B)** Functional annotation clustering of pathways shows that the top-enriched cluster, in overlapping *Gata6* and *Spdef* genes, contains the Wnt pathway as its main function (ES score 2.58). **(C)** GSEA analysis shows, in the overlapping *Gata6* and *Spdef* gene segment*,* APC targets as the second most highly enriched cluster (ES score 1.57).

To gain insight on the relationship between GATA6 and SPDEF in the small intestine, we scanned the overlap of gene targets using publicly available gene profiling data from conditional *Gata6* and *Spdef* knockout mouse intestine [4,9]. Previously, we identified 2564 genes whose expression is altered in ileum by conditional *Gata6* deletion [4]. Network analysis of this gene set indicated an up-regulation of p53 targets and a down-regulation of c-MYC targets (Additional file 1: Figure S1), consistent with a decrease in cellular proliferation. Of the 2564 genes altered by conditional *Gata6* knockout, 83% (2119) were also altered by *Spdef* knockout (Figure 1A), a far greater overlap than would be expected from a similar-sized, randomized allocation of genes (P < 10^-60^, Fisher’s Exact Test). The changes in expression of this subset when *Gata6* is deleted were analyzed by gene set array analysis. Using Database for Annotation, Visualization and Integrated Discovery (DAVID), we conducted functional annotation clustering of all the major pathways (listed in the KEGG, Biocarta and BBID databases). We found that Wnt signaling was the function most likely to be effected (Figure 1B), consistent with regulation of crypt cell proliferation. Using Gene Set Enrichment Analysis (GSEA), a publically available bioinformatics tool that delineates gene expression data for enrichment of pre-defined gene-sets, APC target network was one of the top three networks affected by *Gata6* deletion with a Normalized Enrichment Score (NES) of -1.57 (Figure 1C). This analysis reveals a very strong overlap in gene targets between GATA6 and SPDEF, and suggests that the principal function of this overlap involves cellular proliferation.

### GATA6 regulates *Spdef* gene expression and occupies a locus in the *Spdef* 5′-flanking region in human Caco-2 cells

**Figure 2:**
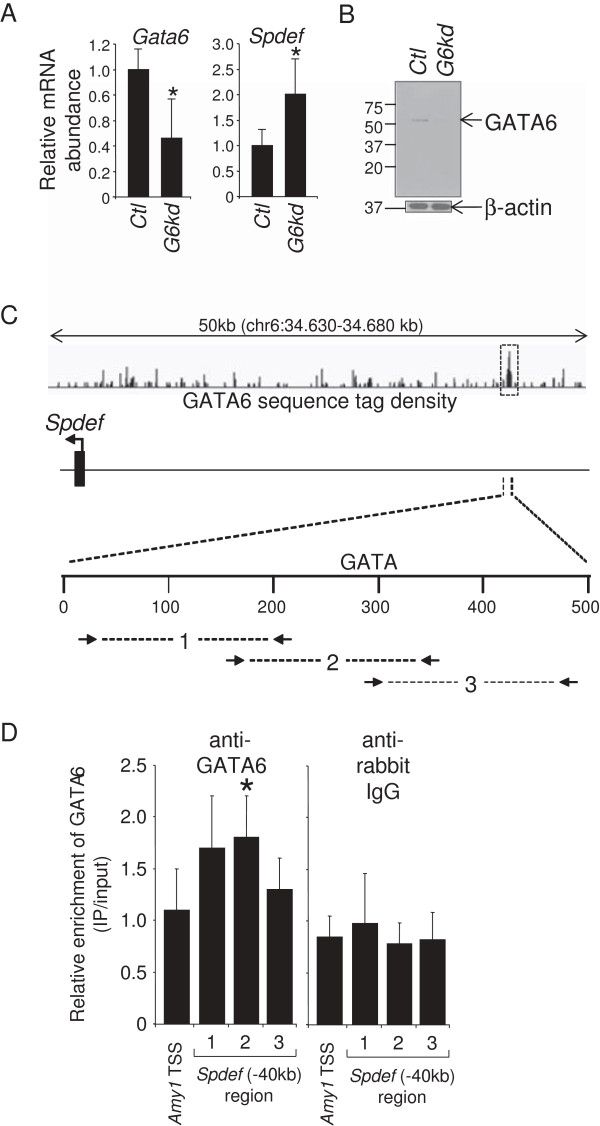
**GATA6 regulates *Spdef *expression and occupies an enhancer in the *Spdef *5′-flanking region in Caco-2 cells. (A)** Quantitative RT-PCR analysis showing reduced expression of *Gata6* mRNA, and enhanced expression of *Spdef* mRNA in Caco-2 cells infected with a lentivirus vector expressing an shRNA for human *Gata6* mRNA (*G6kd*) (mean ± SD, n = 5, *P < 0.05). An shRNA vector for GFP was used as a control (*Ctl*). **(B)** Western analysis showing reduced abundance of GATA6 in *G6kd* Caco-2 cells. Β-actin was used as an internal loading control. **(C)** Schematic representation of the human *Spdef* 5′-flanking region showing GATA6 occupancy at a locus ~40 kb upstream of the transcription start site (TSS). GATA6 sequence tag density is shown as a 'wiggle file’, and a statistically significant GATA6-occupied locus was defined by MACS peak analysis [29] (*dotted box*). **(D)** ChIP assays on chromatin obtained from Caco-2 cells using a GATA6 antibody and three sets of overlapping primers centered on the GATA motif showing increased GATA6 occupancy (mean ± SD, n = 4, *P < 0.05). The salivary amylase-α1a (*Amy-1*) TSS was used as a negative control. ChIP assays using anti-rabbit IgG was used as a control for non-specific immunoprecipitation and primer efficiency.

Conditional *Gata6* deletion in mice produces an up-regulation of *Spdef* gene expression in ileum while knockout or over-expression of *Spdef* has no effect on *Gata6* expression [4], suggesting that *Spdef* is regulated downstream by GATA6. To determine the extent to which this process is conserved, and to further explore the role of GATA6 in regulating *Spdef* gene expression, we characterized the effect of *Gata6* knock-down on *Spdef* gene expression in Caco-2 cells. The Caco-2 cell line is a human colorectal adenocarcinoma-derived cell line that expresses GATA6, but very little GATA4 [11], similar to the ileum. We screened five GATA6 short-hairpin RNA (shRNA) knockdown lentiviral constructs, and found that only one resulted in a statistically significant knockdown of *Gata6* mRNA (55%, P < 0.05, Figure 2A) and a concomitant decrease in GATA6 protein (Figure 2B). *Spdef* mRNA was up-regulated 2-fold in *Gata6* knock-down (*G6kd*) cells (P < 0.05, Figure 2A), demonstrating that the Caco-2 cells model the up-regulation of *Spdef* gene expression observed in conditional *Gata6* knockout mouse ileum [4].

To gain insight on the relationship between GATA6 and *Spdef* gene expression, we examined GATA6 chromatin occupancy at the *Spdef* gene locus. Because we were unable to immunoprecipitate bound chromatin from crosslinked mouse intestinal epithelial cells using existing antisera, we utilized the human Caco-2 intestinal cell culture model in which GATA6 ChIP assays have been performed previously [12]. Using a publicly available GATA6 chromatin immunoprecipitation-high throughput sequencing (ChIP-seq) database in Caco-2 cells [12], we identified a GATA6-occupied *cis*-regulatory region approximately 40 kb upstream of the *Spdef* transcription start site (TSS) that mapped to the *Spdef* gene based on closest distance to any TSS (Figure 2C). To confirm and localize GATA6 occupancy at this locus, we performed GATA6 ChIP assays using a multiple, overlapping primer strategy with amplicons of ~200 bp per primer pair (Figure 2C) centered at the only GATA motif within this region. We found increases of all three amplicons with amplicon from primer set 2 being significantly greater (~70%, P < 0.05, n = 4) than the *Amy1* TSS negative control (Figure 2D). Although our ChIP data reveal a modest increase in enrichment, it is nonetheless statistically significant and thus confirmatory of ChIP-seq data conducted by others [12] (see MACS peak data, Figure 2C). We also conducted an IgG control ChIP assay to show that enrichment with primer set 2 was not due to differences in primer efficiency from that of the *Amy1* TSS, or to non-specific antibody binding (Figure 2D). These data confirm previous ChIP-seq analysis that GATA6 occupies a *cis*-regulatory region that maps to the *Spdef* gene [12], and, together with *Spdef* up-regulation in *G6kd* cells, is consistent with the notion that GATA6 directly represses *Spdef* gene transcription. While mapping of occupancy sites to the nearest TSS is a well recognized method for defining transcription factor targets on a global basis [12,13], direct regulation of *Spdef* gene expression by GATA6 at this site will need to be confirmed using chromosome conformation capture or other techniques [14].

Transcriptional repression is highly complex, especially in eukaryotes, generally involving the recruitment of specific co-repressors and the local modification of histone tails and chromatin structure [15]. GATA factors are well known to mediate gene repression, and the mechanisms are beginning to be understood. GATA4 has been shown to directly repress cardiac genes [16] while GATA1 has been shown to directly repress hematopoietic genes [17,18]. Both GATA4 and GATA1 interact directly with corepressor complexes including the nucleosome remodeling and histone deacetylase (NuRD) complex and the polycomb repressive complex 2 (PRC2) [16], and are necessary for the multiple modifications of histone tails, but the extent to which GATA factors recruit co-repressors and/or modulate histone tails in the small intestine remains to be determined.

### *Spdef* knockout rescues the proliferation defect in conditional *Gata6* knockout mice

We next asked to what extent *Spdef* deletion rescues the conditional *Gata6* knockout phenotype by analyzing single and double *Gata6/Spdef* knockout mice. In previous studies, conditional deletion of *Gata6*[4] or deletion of *Spdef*[9] resulted in greatly diminished levels of *Gata6* or *Spdef* mRNA, respectively, in the intestine that correlated with reduced protein levels and altered intestinal phenotypes. Analysis of mRNA in the present study showed that *Gata6* and *Spdef* mRNAs were both expressed in *Ctl* mice, *Gata6* mRNA was nearly undetectable while *Spdef* mRNA was expressed in *Gata6ΔIE* mice, *Gata6* mRNA was expressed while *Spdef* mRNA was nearly undetectable in *SpdefKO* mice, and both *Gata6* and *Spdef* mRNAs were nearly undetectable in the double knockout (*DKO*) mice (Additional file 1: Figure S2), verifying our models.

**Figure 3:**
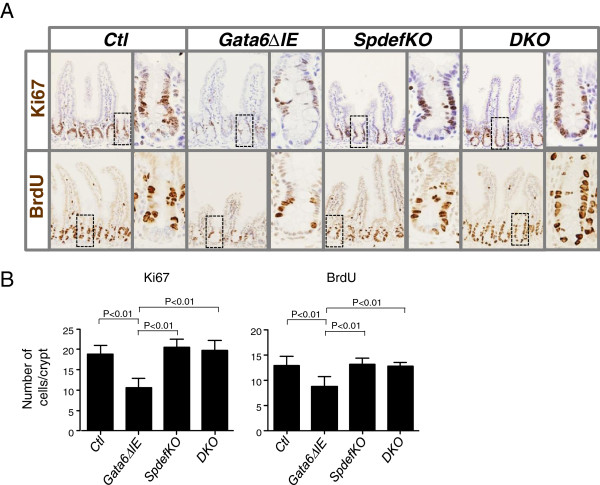
**Crypt cell proliferation is decreased in *Gata6ΔIE *mice. (A)** Immunostaining in ileum for the proliferation markers Ki67 (*top row*) and BrdU (*bottom row*). **(B)** Quantification of Ki67- and BrdU-positive cells/crypt in each group. Cells were counted as described in Methods. *Gata6ΔIE* mice had significantly fewer Ki67- or BrdU-positive cells than each of the other three groups (P < 0.01 in each case), consistent with a decrease in crypt cell proliferation.

Crypt cell proliferation is essential for the continuation of intestinal epithelial renewal. One of the principal phenotypic outcomes of conditional *Gata4/Gata6* deletion is a ~30% reduction in the number of Ki67- and BrdU-positive cells, and a concomitant decrease in villus height and villus epithelial cell number [4] resulting in a reduction in absorptive surface area. To define the role of SPDEF in mediating the decrease in crypt cell proliferation when *Gata6* is conditionally deleted, we stained ileal segments for Ki67 and BrdU (Figure 3A), markers of the non-G_0_ and S-phases of the cell cycle, respectively. The number of Ki67-positive and BrdU-positive crypt cells was significantly reduced ~40% in *Gata6ΔIE* mice (Figure 3B), as previously reported [4], but unchanged in *SpdefKO* mice, as compared to controls. The number of Ki67- and BrdU-positive crypt cells in *DKO* mice was also similar to controls, indicating that the additional deletion of *Spdef* rescues the decrease in crypt cell proliferation observed in *Gata6ΔIE* mice. These data support the notion that GATA6 maintains crypt cell proliferation by down-regulating *Spdef* gene expression.

### SPDEF does not regulate the secretory cell differentiation function of GATA6

**Figure 4:**
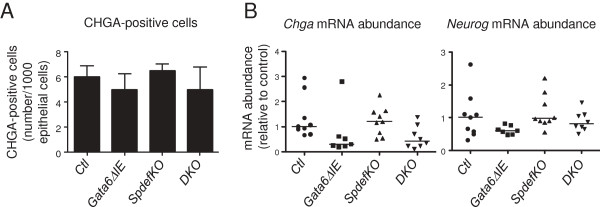
**Enteroendocrine cell allocation is decreased in *Gata6ΔIE *and *DKO *mice. (A)** Quantification of CHGA-positive cells in each group. Sections of ileum were stained for CHGA, and the number of positive cells/1000 epithelial cells was determined as described in Methods. **(B)***Chga* and *Neurog3* mRNA abundance in each group. *Gata6ΔIE* and *DKO* mice had generally lower numbers of CHGA-positive cells and *Chga* and *Neurog3* mRNA abundances than *Ctl* mice, consistent with a decrease in enteroendocrine lineage allocation.

Previously, conditional deletion of *Gata6* in the ileum resulted in a 25-40% reduction in the number of chromogranin A (CHGA)-positive cells, and in the mRNA abundances of *Chga* and *Neurog3*[4], consistent with a decrease in enteroendocrine lineage commitment. Though not statistically significant, we found a 15-50% reduction in the number of CHGA-positive cells (Figure 4A), and in the mRNA abundances of *Chga* and *Neurog3* (Figure 4B) in the *Gata6ΔIE* mice, in general agreement with our previous study [4]. We further found that although the pattern for these three measurements in the *SpdefKO* mice was similar to that in *Ctl* mice, consistent with previous data for distal intestine [9], the pattern in the *DKO* mice was similar to that in the *Gata6ΔIE* mice (Figure 4A and B). Together, these data show that enteroendocrine cell commitment is reduced in both *Gata6ΔIE* and *DKO* mice, indicating that GATA6 promotes enteroendocrine cell commitment independently of SPDEF.

**Figure 5:**
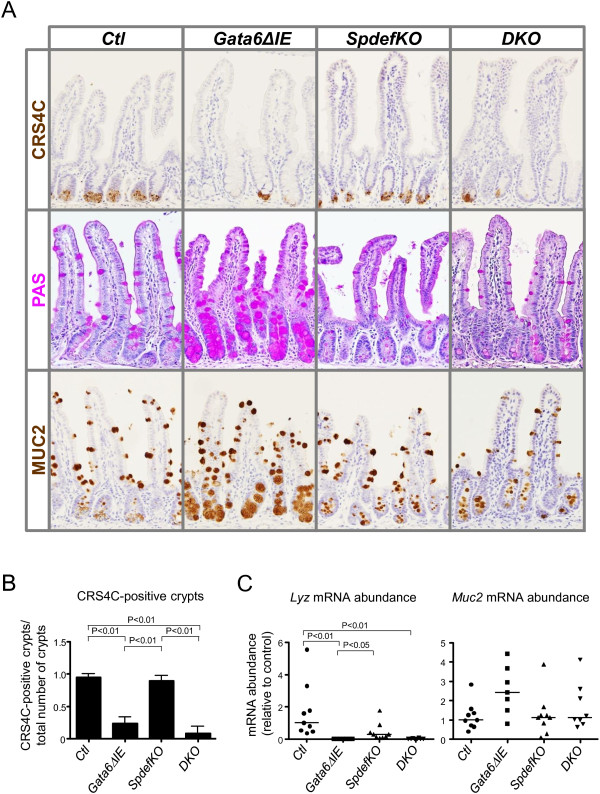
**Paneth cells are decreased in *Gata6ΔIE *and *DKO *mice. (A)** Immunostaining in ileum for CRS4C (*first row*) and MUC2 (*third row*), and chemical staining using the PAS reaction (*second row*). **(B)** Quantification of CRS4C-positive cells in each group. Sections of ileum were stained for CRS4C, and the number of positive cells/crypt cross section was determined as described in Methods. **(C)***Lyz* and *Muc2* mRNA abundance in each group. *Gata6ΔIE* and *DKO* mice had significantly lower numbers of CRS4C-positive cells and *Lyz* mRNA abundance than *Ctl* mice, consistent with a decrease in mature Paneth cells.

Previously, we also showed that conditional *Gata6* deletion resulted in a transformation of Paneth cells into Mucin-2 (MUC2)-enriched goblet-like cells at the base of crypts [4]. In the present study, immunostaining for CRS4C, a Paneth-specific marker, was slightly reduced in *SpdefKO* mice as compared to controls, consistent with previous data [9], but was greatly reduced in both *Gata6ΔIE* and *DKO* mice as compared to controls (Figure 5A). The number of crypt cross-sections with at least one CRS4C-positive cell was nearly 100% in *Ctl* and *SpdefKO* mice, but was less than 30% in both *Gata6ΔIE* and *DKO* mice (P < 0.01 for each, Figure 5B). The mRNA abundance for lysozyme (*Lyz*), another Paneth marker, was not significantly different between *Ctl* and *SpdefKO* mice, but was significantly reduced in *Gata6ΔIE* and *DKO* mice as compared to controls (P < 0.01 for each, Figure 5C). PAS staining was greatly increased in crypts of *Gata6ΔIE* mice, as previously shown [4], noticeably reduced in *SpdefKO* mice, especially in villus goblet cells, as previously shown [9], and similar to controls in *DKO* mice (Figure 5A). MUC2 immunostaining was greatly enriched in crypts of *Gata6ΔIE* mice, consistent with our previous report [4], but similar to controls in *SpdefKO* and *DKO* mice (Figure 5A). Generally consistent with this observation, *Muc2* mRNA abundance in *Gata6ΔIE* mice was 2.5-fold higher as compared to controls, though not statistically significant, whereas *Muc2* mRNA abundances in *SpdefKO* and *DKO* mice were similar to controls (Figure 5C). These data indicate that the additional deletion of *Spdef* did not rescue the decrease in the terminal differentiation of Paneth cells observed in *Gata6ΔIE* mice, but did at least partially prevent their conversion to MUC2-enriched goblet-like cells. Thus, SPDEF is not necessary for the GATA-mediated differentiation of Paneth cells, but does function in their default differentiation into goblet-like cells in the absence of *Gata6*. This is consistent with the general function of SPDEF to promote the differentiation of goblet cells [9,10].

In spite of the common secretory cell phenotypes in *Gata6* deletion and *Spdef* over-expression models [4,10], our data show that *Spdef* deletion did not rescue the *Gata6* knockout defects in enteroendocrine lineage commitment or in Paneth cell differentiation. Closer scrutiny suggests that the secretory cell phenotypes in the *Gata6* deletion and *Spdef* over-expression models are not identical. While the decline in enteroendocrine and Paneth cells, and accumulation of goblet cells, appear similar, the underlying alteration in Paneth and goblet cell differentiation is different. Conditional *Gata6* deletion has no effect on the normal commitment and differentiation of goblet cells on villi but reveals a conversion of Paneth cells at the base of crypts into a goblet-like cell type. These cells do not express defensins, but express abundant *Muc2;* they also express abundant *Sox9* and *Ephb3*[4], indicating that they are committed and targeted Paneth cells. On the other hand, conditional *Spdef* over-expression results in a generalized increase in goblet cells at the expense of all other epithelial cell types [10]. Hence, these mice show a decline in Paneth cell specification rather than a defect in the terminal differentiation of committed Paneth cells, as observed in our conditional *Gata6* knockout model.

### SPDEF does not regulate the absorptive enterocyte gene expression function of GATA6

**Figure 6:**
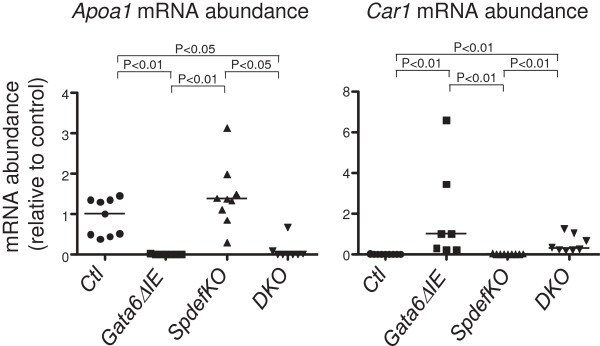
**Absorptive enterocyte gene expression is altered in *Gata6ΔIE *and *DKO *mice. ***Gata6ΔIE* and *DKO* mice had significantly lower *Apoa1*, and significantly higher *Car1* mRNA abundances than *Ctl* mice.

We next examined whether *Spdef* loss affected GATA6-dependent absorptive enterocyte gene expression. Conditional *Gata6* deletion resulted in a down-regulation of specific absorptive enterocyte genes in ileum that include lipid transporters and apolipoproteins, and an up-regulation of genes in absorptive enterocytes normally not expressed or expressed at low levels in small intestine, but expressed at high levels in colon [4]. Using marker genes for these two patterns, apolipoprotein A1 (*Apoa1*) and carbonic anyhdrase 1 (*Car1*), respectively, we found that their mRNA abundances were down-regulated and up-regulated, respectively, in *Gata6ΔIE* mice (Figure 6), consistent with our previous findings [4]. *Apoa1* and *Car1* mRNA abundance in *SpdefKO* mice was similar to controls, whereas that in *DKO* mice was similar to *Gata6ΔIE*, indicating that SPDEF does not play a role in mediating the conditional *Gata6* knockout alteration in absorptive enterocyte gene expression.

GATA6 is expressed in crypts and in mature absorptive enterocytes [4,5], whereas SPDEF is expressed in secretory progenitors, and in goblet and Paneth cells, but is not expressed in mature absorptive enterocytes [9,10]. While it is possible that SPDEF could instruct gene expression in absorptive enterocytes through a process that originates in progenitors early in the differentiation process, we found that SPDEF did not regulate the GATA6 targets studied here. As in the GATA4-specific pathway, in which GATA4 regulates its targets in mature absorptive enterocytes on villi rather than in crypt progenitors [19], we believe that GATA6 also regulates absorptive enterocyte gene expression within mature absorptive enterocytes on villi.

## Conclusion

**Figure 7:**
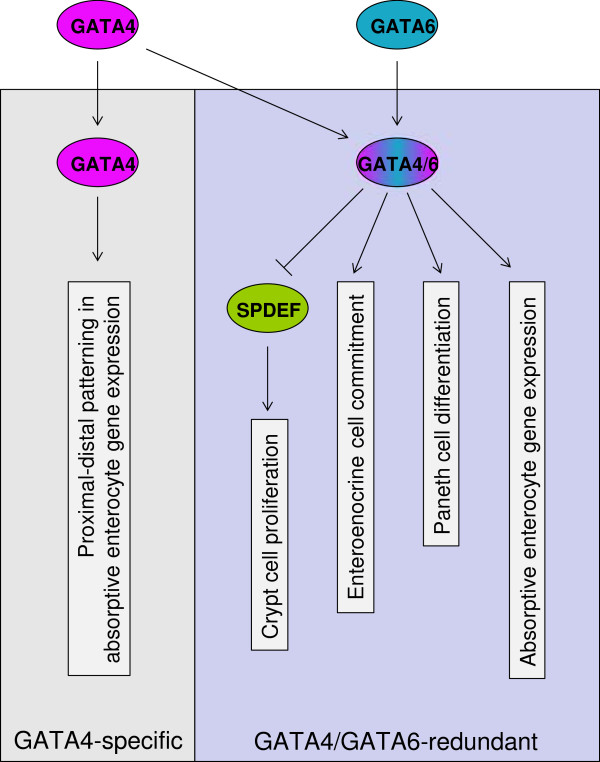
**Model for the known GATA pathways in mature small intestine, and the placement of SPDEF within those pathways.** GATA4 (*purple*), but not GATA6 (*blue*), activates and represses a subset of absorptive enterocyte genes (GATA4-specific), whereas either GATA4 or GATA6 (*purple/blue*) promote crypt cell proliferation, enteroendocrine lineage commitment, Paneth cell differentiation, and absorptive enterocyte gene expression (GATA4/GATA6-redundant). GATA4/GATA6 promotes the crypt cell proliferation function by repressing the gene encoding SPDEF (green).

Previously, we defined two fundamental pathways of GATA regulation in the small intestine, one mediated exclusively by GATA4 (GATA4-specific pathway), and one regulated by GATA4 or GATA6 (GATA4/GATA6-redundant pathway) (Figure 7). In the GATA4-specific pathway, GATA4, but not GATA6, activates and represses a subset of absorptive enterocyte genes, and by virtue of its expression in the proximal 85% of small intestine and lack of expression in distal ileum [7], distinguishes proximal intestinal from distal ileal gene expression and function [5,7,8]. For the GATA4/GATA6-redundant pathway, either GATA4 or GATA6 (which is expressed throughout the small intestine, including distal ileum) promote intestinal epithelial renewal by supporting crypt cell proliferation, enteroendocrine lineage commitment, Paneth cell differentiation, and absorptive enterocyte gene expression [4]. Here, we show that the maintenance of crypt cell proliferation function of the intestinal GATA4/GATA6-redundant pathway is dependent on SPDEF (Figure 7). Specifically, our data support the notion that GATA4/GATA6 promotes crypt cell proliferation by directly repressing *Spdef* gene expression.

GATA6 has been suggested to be oncogenic in multiple cancers and pre-cancerous lesions, including Barrett’s esophagus, gastric cancer, pancreatic cancer, and colon cancer [20]. *Gata6* expression is up-regulated in colon cancer epithelial cells [21-23], as well as in non-malignant cells along the stromal margins in human colorectal cancer [22], but it should be noted that it has not been determined whether this is a correlative, causative or protective event. Recently, Noah et al. [24] showed that SPDEF functions as a colorectal tumor suppressor. In colorectal tumors from patients, loss of SPDEF was observed in approximately 85% of tumors and correlated with progression from normal tissue, to adenoma, to adenocarcinoma. Further, SPDEF inhibited the expression of β-catenin-target genes in mouse colon tumors, and interacted with β-catenin to block its transcriptional activity in colorectal cancer cell lines, resulting in lower levels of cyclin D1 and c-MYC. Pathway analysis showed that *Gata6* deletion also modulated c-MYC targets (Additional file 1: Figure S1) and that the GATA6/SPDEF overlapping gene set likely functions in Wnt signaling (Figure 1). Thus, our data here suggest that the possible tumorigenic effects of GATA6 could be mediated by its repression of *Spdef* gene expression.

## Methods

### Analysis of publicly available gene profiling data

From the National Center for Biotechnology Information (NCBI) website (http://www.ncbi.nlm.nih.gov/), we downloaded gene profiling data from the ileum of conditional *Gata6* knockout mice (GSE22416) [4], and from the small intestine of *Spdef-/-* mice (GSE14892) [9]. Data analysis was performed using Cistrome (http://www.cistrome.org), a flexible bioinformatics workbench with an analysis platform for ChIP-seq and gene expression microarray analysis [25]. Significant over-representation of overlapping target genes was determined by the Fisher’s Exact Test. Gene ontology analysis and pathway analysis was conducted using Database for Annotation, Visualization and Integrated Discovery (DAVID) [26]. Gene set enrichment analysis was performed by using publicly available Gene Set Enrichment Analysis (GSEA) tools v2.3 from the Broad Institute [27].

### Cell culture and lentiviral infection

Lentiviral infection was conducted as previously described [28]. 293 T and Caco-2 cells were cultured in Dulbecco’s modified Eagle’s medium (DMEM; Mediatech, Inc., Manassas, VA) containing glucose (4.5 g/liter), l-glutamine, and sodium pyruvate. The media was supplemented with 10% fetal bovine serum (Atlanta Biologicals, Lawrenceville, GA) and penicillin (100 U/ml)-streptomycin (1 mg/ml) (Sigma Chemical Company, St. Louis, MO). Both cell lines were maintained in 5% CO_2_ at 95% relative humidity and 37°C. Media were replaced every 2 to 3 days. To produce lentivirus for the knockdown of *Gata6*, 293 T cells were plated at 10^5^ cells/ml on 6-cm plates and transfected after 24 h with 1 μg of vesicular stomatitis virus glycoprotein G (VSV-G) envelope-expressing plasmid pMD.G, 1 μg of pCMV-dR8.91 (Delta 8.9) plasmid containing *gag*, *pol*, and *rev* genes, and 1 μg of shRNA-pLKO.1 plasmids expressing a knockdown short hairpin RNA (shRNA) (Sigma) for green fluorescent protein (GFP) (used as a control [*Ctl*]) (SHC005) or human *Gata6* (*G6kd*) using 6 μl of FuGENE 6 reagent (Roche Diagnostic Corporation, Indianapolis, IN). A total of five human *Gata6* knockdown constructs were screened, and only one (TRCN0000005392) was subsequently found to reduce *Gata6* mRNA significantly. Media from the transfected 293 T cells were changed 16 h after transfection. Culture media containing lentiviral particles were collected and filtered (0.45 μm pore size) 48 h after transfection and transferred immediately to Caco-2 cells plated at 30% confluence to infect for 2 h using 0.5 μl of polybrene/ml. Infection was repeated the next day followed by selection of infected cells using 4 μg/ml puromycin. Infected cells were kept under conditions of selection until the day of harvesting. Trypsinized cells were homogenized using a QIA shredder (Qiagen, Inc., Valencia, CA), and RNA was isolated as described above for mouse tissue. Nuclear extracts were isolated as described previously [11]. *Gata6* knockdown was determined by quantitative reverse transcriptase- (qRT-) PCR for human *Gata6* (see Additional file 1: Figure S3A for primers) and by Western blot analysis as previously described [5] using rabbit anti-GATA6 (Cell Signaling Technology, Inc, Danvers, MA; Cat. No. 4253S).

### Chromosomal immunoprecipitation (ChIP) assays

Using a publicly available GATA6 chromatin immunoprecipitation-high throughput sequencing (ChIP-seq) database in Caco-2 cells [12], sequences were mapped to reference genome *Homo Sapiens* build 18 (HG18) using ELAND tools, allowing 0 to 2 mismatches (Illumina), and binding peaks were identified by model-based analysis of ChIP-seq (MACS) [29] using default parameters and *P* value cutoffs of 10^-10^.

Chromosomal immunoprecipitation (ChIP) assays were conducted as previously described [28]. Caco-2 cells were incubated in DMEM containing 1% formaldehyde (Fisher Scientific, Pittsburgh, PA) for 10 min at 37°C. The cells were washed 2 times with PBS, scraped, and resuspended in lysis buffer (50 mM Tris-Cl [pH 8.1], 10 mM EDTA, 1% sodium dodecyl sulfate [SDS]) containing protease inhibitor cocktail and PMSF (10 μl/ml). The samples were sonicated to obtain chromatin fragments of between 400 and 1,000 bp. Sonicated samples were resuspended in ChIP dilution buffer (1% Triton X-100, 2 mM EDTA, 150 mM NaCl, 20 mM Tris–HCl, pH 8.1) and incubated overnight at 4°C with Dynabeads beads (Life Technologies, Grand Island, NY) conjugated with rabbit anti-GATA6 (Cell Signaling; Cat. No. 4253) or rabbit IgG (negative control) (Millipore, Temecula, CA; Cat. No. PP64B). The IP samples were washed 6 times with radioimmunoprecipitation (RIPA) buffer (50 mM HEPES [pH 7.6], 0.5 M LiCl, 1 mM EDTA, 1% Nonidet P-40, 0.7% sodium deoxycholate), and the DNA was recovered by reverse cross-linking in 1% SDS–0.1 M NaHCO_3_ for 7 h at 65°C. DNA was purified using a QIAquick PCR purification kit (Qiagen) and quantified by Picogreen (Life Technologies). One nanogram of DNA was used per qPCR reaction using primers shown in Additional file 1: Figure S3B.

### Mice

Previously established and confirmed *Gata6*^*loxP/loxP*^[30], *Spdef-/-*[9], and transgenic Villin*Cre*ER^T2^[31] mice were used in this study to produce four groups of mice, including controls (*Ctl*), conditional *Gata6* deletion in the intestinal epithelium (*Gata6ΔIE*), germline *Spdef* knockout (*SpdefKO*), and double *Gata6/Spdef* knockout (*DKO*). The genotypes of each group are as follows:

*Ctl*: *Gata6*^*loxP/loxP*^, *Spdef +/-*, Villin*Cre*ER^T2^-negative

*Gata6ΔIE: Gata6*^*loxP/loxP*^, *Spdef +/-*, Villin*Cre*ER^T2^-positive

*SpdefKO: Gata6*^*loxP/loxP*^, *Spdef -/-*, Villin*Cre*ER^T2^-negative

*DKO*: *Gata6*^*loxP/loxP*^, *Spdef -/-*, Villin*Cre*ER^T2^-positive

DNA was extracted from tail biopsies, and genotypes were determined by semiquantitive polymerase chain reaction (PCR) using previously validated primers (Additional file 1: Figure S3C). Male and female mice four weeks of age were treated with a single dose of tamoxifen (Sigma) (0.1 ml, 10 mg/ml; dissolved in ethanol/sunflower oil = 1:9 [vol/vol]) daily for five consecutive days as described [5], followed by a single dose two weeks later (see timeline, Additional file 1: Figure S4). Mice were killed and tissue was collected 28 days after the start of tamoxifen treatment. Bromodeoxyuridine (BrdU, 0.1 ml of 10 mg/ml) was injected two hours before dissection. Approval was obtained from the Institutional Animal Care and Use Committee.

### Tissue isolation and processing

Mice were anesthetized for dissection as previously described [28]. The most distal 1.0 cm segment of small intestine adjacent to the ileocecal valve was snap frozen for RNA isolation, and the 6 cm segment proximal to that was removed and transferred to a glass plate on wet ice and prepared for sectioning. This segment was flushed with ice cold 4% paraformaldehyde (PFA) in PBS, cut longitudinally, pinned open onto paraffin wax in a petri dish filled with 4% PFA in PBS with the epithelium facing upward. After a 5 min incubation period, the pins were removed and the proximal end was grasped with forceps, rolled with the epithelium facing outwards, and a pin was inserted transversely to secure the roll in place. The roll was placed in 10 ml of 4% PFA in PBS and mixed gently on a tube rotator for 16–18 hr at 4°C. The PFA was decanted and the tissue was washed in PBS 3 times for 20 min each at 4°C, and then dehydrated in 70% ethanol. Tissue was processed at the Rodent Histopathology Core at the Dana Farber/Harvard Cancer Center (Boston, MA) for paraffin embedding and sectioning. Selected slides were stained using the periodic acid Schiff (PAS) reaction.

### RNA isolation and gene expression analysis

RNA was isolated using the RNeasy kit (Qiagen), and mRNA abundances were determined by qRT-PCR as previously described [4], using validated primer pairs (Additional file 1: Figure S3A). Glyceraldehyde-3-phosphate dehydrogenase (GAPDH) mRNA abundance was measured for each sample and used to normalize the data. Data were expressed relative to the median value of control ileum. A minimum of five mice in each group was analyzed.

### Immunohistochemistry

Tissue sections were immunostained as previously described [28]. Primary antibodies included rabbit anti-Ki67 (Thermo Fisher Scientific, Inc., Fremont, CA; Cat. No. RM-9106-S1) (1:200), mouse anti-BrdU (Thermo; Cat. No. MS-1058-PO) (1:250), goat anti-cryptdin related sequence 4C (CRS4C) (gift from Dr. A. J. Ouellette, University of Southern California, Los Angeles, CA) [32] (1:2000), and rabbit anti-MUC2 (Santa Cruz Biotechnology, Inc, Santa Cruz, CA; Cat. No. sc15334) (1:100). Secondary antibodies included biotinylated donkey anti-rabbit IgG, donkey anti-goat IgG, and donkey anti-mouse IgG (all from Vector Labs, Burlingame, CA). Biotinylated antibodies were linked to avidin-horseradish peroxidase conjugates (Vector Labs), visualized using 3,3′-diamino benzidine (Sigma) for 2 to 5 min, and lightly counterstained with hematoxylin.

### Cell counting

The total number of Ki67-, BrdU-, or CRS4C-positive cells in crypts was determined as the total number per crypt. Only well oriented crypts with the epithelial layer on at least one side continuous with the villus epithelial layer were counted, and a minimum of 6 crypts per slide were analyzed. The average number of CHGA–positive cells was expressed as a fraction of total epithelial cells (villi and crypts) from a minimum of 5000 epithelial cells per slide, with equal representation of crypts and villi. All determinations were blinded and conducted on a minimum of 5 animals per group.

### Statistical analyses

In Caco-2 cells, qRT-PCR data was compared by the student’s t-test and ChIP data were compared by the analysis of variance (ANOVA) followed by the Tukey-Kramer multiple comparison test. In mice, mRNA measurements had unequal variances across groups requiring nonparametric statistics, and were thus compared by the Kruskal-Wallis test followed by the Dunn multiple comparison test, and presented as individual data points and medians. Cell count determinations had equal variances across groups allowing parametric statistics, and were thus compared by ANOVA followed by the Tukey-Kramer multiple comparison test, and presented as mean ± SD. Differences were considered statistically significant at a *P*-value of less than 0.05.

## Competing interests

The authors declare no competing interest.

## Authors’ contributions

BEA, LATMV, ES and HB performed and interpreted the Caco-2 cell culture experiments. KAS performed and interpreted the in vivo mouse experiments. BEA, KAS and SDK contributed to the study design and interpretation of results, and the writing of the manuscript. All authors read and approved the final manuscript.

## Supplementary Material

Additional file 1: Figure S1 Intestinal *Gata6* deletion alters gene networks controlling cell proliferation in the mature ileum. Network analyses on microarray data of *Ctl* and *Gata6ΔIE* ileum (n=3 in each group) revealed (A) an increase in targets of the tumor surpressor gene p53, and (B) a decrease in targets of the proto-oncogene c-MYC. (C) Legend defining symbols used in the networks. Arrows indicate the direction of the interaction. Red circles = up-regulated transcripts; Blue circles = down-regulated transcripts. Differentially expressed transcripts were determined at the 5% FDR level using Significance Analysis of Microarrays (SAM) and interaction networks were developed from the differentially expressed transcripts using Metacore. **Figure S2**. *Gata6* and *Spdef* mRNA abundance in ileum in each group of mice. *Gata6ΔIE* and *DKO* mice had significantly lower *Gata6* mRNA abundances than *Ctl* and *SpdefKO* mice. *SpdefKO* and *DKO* mice had significantly lower *Spdef* mRNA abundances than *Ctl* and *Gata6ΔIE* mice. **Figure S3**. Primers used for: (A) qRT-PCR, (B) ChIP assays, and (C) genotyping. **Figure S4**. Timeline for study. Mice 4 wks of age were given Tamoxifen as indicated (*black circle*) beginning on Day 0, and an injection of BrdU 2 hr before tissue collection at Day 28. Click here for file
