# Supplementary material for: Spdef deletion rescues the crypt cell proliferation defect in conditional Gata6 null mouse small intestine
Source: BMC Mol Biol. 2014 Jan 28;15:3. doi: 10.1186/1471-2199-15-3 (PMC3917371; doi:10.1186/1471-2199-15-3)
Supplement: Additional file 1: Figure S1 — Intestinal Gata6 deletion alters gene networks controlling cell proliferation in the mature ileum. Network analyses on microarray data of Ctl and Gata6ΔIE ileum (n=3 in each group) revealed (A) an increase in targets of the tumor surpressor gene p53, and (B) a decrease in targets of the proto-oncogene c-MYC. (C) Legend defining symbols used in the networks. Arrows indicate the direction of the interaction. Red circles = up-regulated transcripts; Blue circles = down-regulated transcripts. Differentially expressed transcripts were determined at the 5% FDR level using Significance Analysis of Microarrays (SAM) and interaction networks were developed from the differentially expressed transcripts using Metacore. Figure S2. Gata6 and Spdef mRNA abundance in ileum in each group of mice. Gata6ΔIE and DKO mice had significantly lower Gata6 mRNA abundances than Ctl and SpdefKO mice. SpdefKO and DKO mice had significantly lower Spdef mRNA abundances than Ctl and Gata6ΔIE mice. Figure S3. Primers used for: (A) qRT-PCR, (B) ChIP assays, and (C) genotyping. Figure S4. Timeline for study. Mice 4 wks of age were given Tamoxifen as indicated (black circle) beginning on Day 0, and an injection of BrdU 2 hr before tissue collection at Day 28. [file 1471-2199-15-3-S1.pdf]

Fig. S1

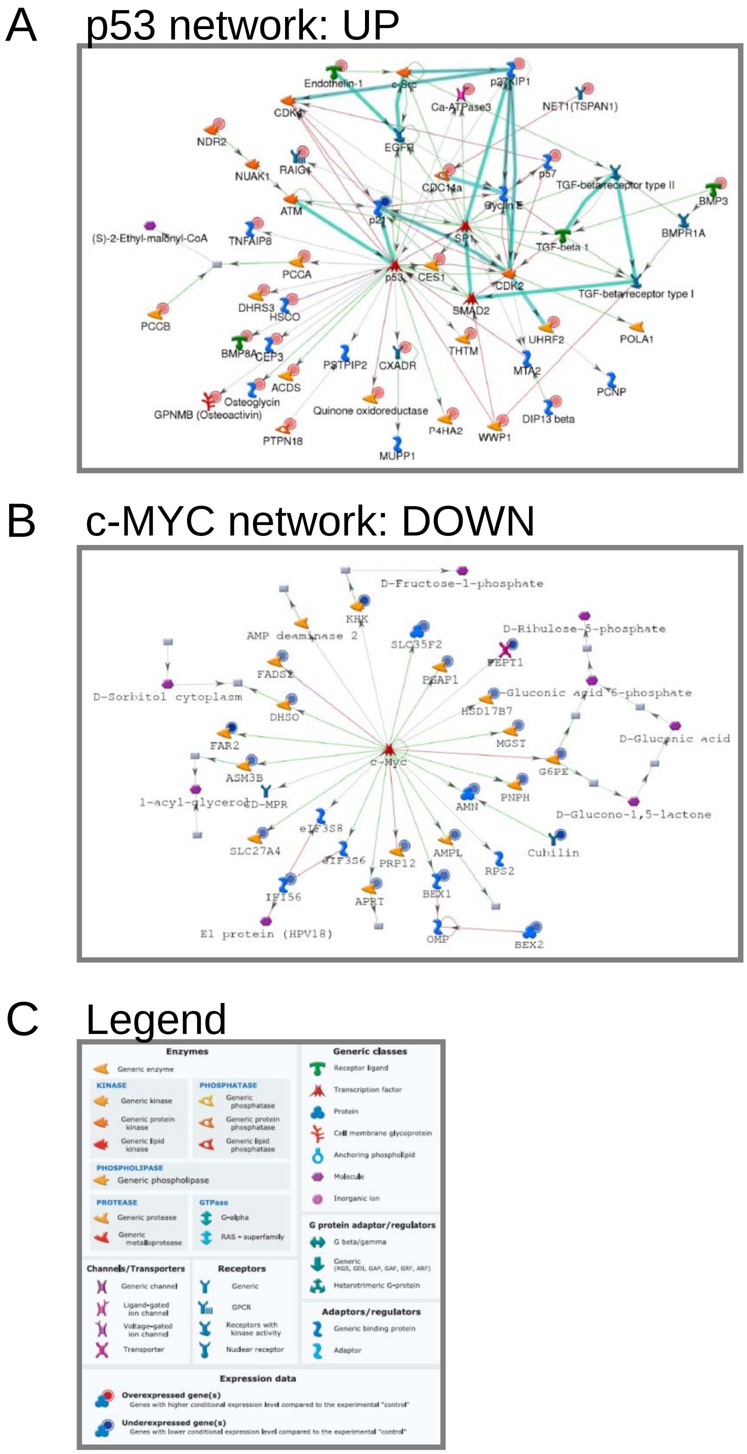

Fig. S1. Intestinal *Gata6* deletion alters gene networks controlling cell proliferation in the mature ileum. Network analyses on microarray data of *Ctl* and *Gata6ΔIE* ileum (n=3 in each group) revealed (A) an increase in targets of the tumor surpressor gene p53, and (B) a decrease in targets of the proto-oncogene c-MYC. (C) Legend defining symbols used in the networks. Arrows indicate the direction of the interaction. Red circles = up-regulated transcripts; Blue circles = down-regulated transcripts. Differentially expressed transcripts were determined at the 5% FDR level using Significance Analysis of Microarrays (SAM) and interaction networks were developed from the differentially expressed transcripts using Metacore.

Fig. S2

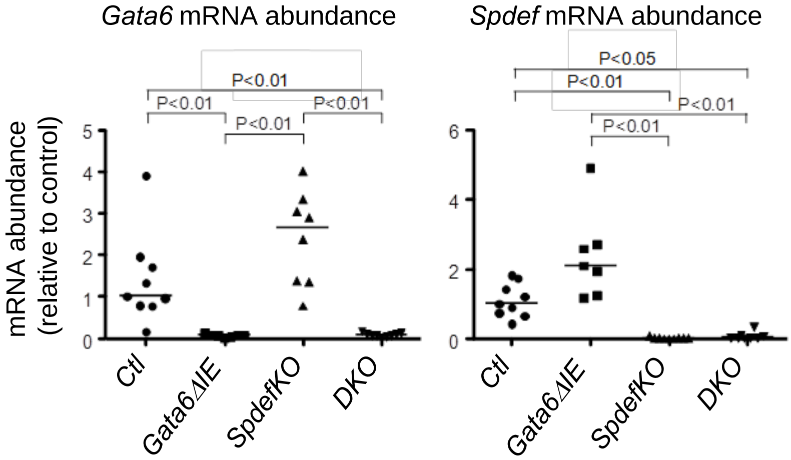

Fig. S2. *Gata6* and *Spdef* mRNA abundance in ileum in each group of mice. *Gata6ΔIE* and *DKO* mice had significantly lower *Gata6* mRNA abundances than *Ctl* and *SpdefKO* mice. *SpdefKO* and *DKO* mice had significantly lower *Spdef* mRNA abundances than *Ctl* and *Gata6ΔIE* mice.

Fig. S3

A qRT-PCR primers

hGata6:	Sense:	5’-GACTTGCTCTGGTAATAG-3’
	Antisense:	5’-CTGTAGGTTGTGTTGTGG-3’
hSpdef:	Sense:	5’-AACATCACCGCAGATCCC-3’
	Antisense:	5’-CGGTATTGGTGCTCTGTC-3’
hGapdh:	Sense:	5’-AAGTATGACAACAGCCTC-3’
	Antisense:	5’-ATGAGTCCTTCCACGATA-3’
mGata6:	Sense:	5’-CAGCAAGCTGTTGTGGTC-3’
	Antisense:	5’-GTCTGGTACATTTCCTCCG-3’
mSpdef:	Sense:	5’-CAACAAGGAGAAAGGCATCTT-3’
	Antisense:	5’-GCGGCTTAGTTTATCATAGTTCA-3’
mChga:	Sense:	5’-CACAGCCACCAATACC-3’
	Antisense:	5’-TCTTCCTCCTCCTCTTC-3’
mNeurog3:	Sense:	5’-CTCAGCAAACAGCGAAGAAG-3’
	Antisense:	5’-GGGAAGGTGGGCAGGAC-3’
mLyz:	Sense:	5’-ACTCCTCCTTGCTTTCTGTC-3’
	Antisense:	5’-GTCGGTGCTTCGGTCTC-3’
mMuc2:	Sense:	5’-AACTACCACTGTGATGCCAATG-3’
	Antisense:	5’-ACAATGTTGATGCCAGACTCG-3’
mApoa1:	Sense:	5’-GGACTTCTGGGATAACCT-3’
	Antisense:	5’-GCACCTTCTGTTTCACTT-3’
mCar1:	Sense:	5’-AACAGAATTATGTCAGTGCTAA-3’
	Antisense:	5’-AGAGAATGAATCACTTAGTTGTAA-3’
mGapdh:	Sense:	5’-GCCTTCCGTGTTCCTACCC-3’
	Antisense:	5’-TGCCTGCTTCACCACCTTC-3’

B ChIP primers

hSpdef (-40 kb), region 1:		
	Sense:	5’-AAAGGTCTTCCTCCAAAATG-3’
	Antisense:	5’-CAAAGCAGTAAGACTTGGAG-3’
hSpdef (-40 kb), region 2:		
	Sense:	5’-CAGTTTGAATCCATCCTCC-3’
	Antisense:	5’-CCTATCTTCAAAGCATTTACTATCT-3’
hSpdef (-40 kb), region 3:		
	Sense:	5’-CTATGGATTGGTCACATCTAC-3’
	Antisense:	5’-GTTTGAGGGTCTTGAACTT-3’
Amy1 TSS:	Sense:	5’-GGAAAATAAAGGGTTGGAGC-3’
	Antisense:	5’-AGTGAATCATGTCAGTATAACAA-3’

C Genotype primers

Gata6:	Sense:	5’-GTGGTTGTAAGGCGGTTTGT-3’
	Antisense:	5’-ACGCGAGCTCCAGAAAAAGT-3’
Cre:	Sense:	5’-CGTATAGCCGAAATTGCCAG -3’
	Antisense:	5’-CAAAACAGGTAGTTATTCGG-3’
Spdef:	WTSense:	5’-CCCACCTCCTATGTCAGCCATGGC-3’
	Antisense:	5’-CAATCCTGTACCATATCTGGCATGG-3’
	KO Sense:	5’-GCATCGCATTGTCTGAGTAGGTGTCA-3’

Fig. S3. Primers used for: (A) qRT-PCR, (B) ChIP assays, and (C) genotyping.

Fig. S4

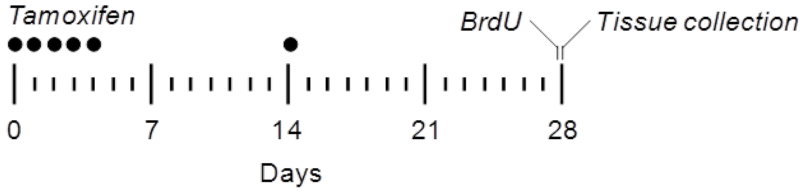

Fig. S4. Timeline for study. Mice 4 wks of age were given Tamoxifen as indicated (*black circle*) beginning on Day 0, and an injection of BrdU 2 hr before tissue collection at Day 28.
